# Mesenchymal stem cell-derived microvesicles improve intestinal barrier function by restoring mitochondrial dynamic balance in sepsis rats

**DOI:** 10.1186/s13287-021-02363-0

**Published:** 2021-05-26

**Authors:** Danyang Zheng, Henan Zhou, Hongchen Wang, Yu Zhu, Yue Wu, Qinghui Li, Tao Li, Liangming Liu

**Affiliations:** State Key Laboratory of Trauma, Burns and Combined Injury, Shock and Transfusion Department, Research Institute of Surgery, Daping Hospital, Army Medical University, Daping, Chongqing, 400042 People’s Republic of China

**Keywords:** Mesenchymal stem cell-derived microvesicles, Sepsis, Intestinal barrier function, mfn2, PGC-1α, Mitochondrial dynamic balance

## Abstract

**Background:**

Sepsis is a major cause of death in ICU, and intestinal barrier dysfunction is its important complication, while the treatment is limited. Recently, mesenchymal stem cell-derived microvesicles (MMVs) attract much attention as a strategy of cell-free treatment; whether MMVs are therapeutic in sepsis induced-intestinal barrier dysfunction is obscure.

**Methods:**

In this study, cecal ligation and puncture-induced sepsis rats and lipopolysaccharide-stimulated intestinal epithelial cells to investigate the effect of MMVs on intestinal barrier dysfunction. MMVs were harvested from mesenchymal stem cells and were injected into sepsis rats, and the intestinal barrier function was measured. Afterward, MMVs were incubated with intestinal epithelial cells, and the effect of MMVs on mitochondrial dynamic balance was measured. Then the expression of mfn1, mfn2, OPA1, and PGC-1α in MMVs were measured by western blot. By upregulation and downregulation of mfn2 and PGC-1α, the role of MMVs in mitochondrial dynamic balance was investigated. Finally, the role of MMV-carried mitochondria in mitochondrial dynamic balance was investigated.

**Results:**

MMVs restored the intestinal barrier function by improving mitochondrial dynamic balance and metabolism of mitochondria. Further study revealed that MMVs delivered mfn2 and PGC-1α to intestinal epithelial cells, and promoted mitochondrial fusion and biogenesis, thereby improving mitochondrial dynamic balance. Furthermore, MMVs delivered functional mitochondria to intestinal epithelial cells and enhanced energy metabolism directly.

**Conclusion:**

MMVs can deliver mfn2, PGC-1α, and functional mitochondria to intestinal epithelial cells, synergistically improve mitochondrial dynamic balance of target cells after sepsis, and restore the mitochondrial function and intestinal barrier function. The study illustrated that MMVs might be a promising strategy for the treatment of sepsis.

## Introduction

Sepsis is a severe host response disorder to infection, which is an important cause of multiple organ dysfunction and a common clinical severe disease [1, 2]. According to epidemiological data in 2016, the total number of sepsis patients was 31.5 million in the globe, and the number has rapidly risen to 48.9 million in 2020, which suggested that sepsis was still a huge burden for global health [1–3]. The intestinal barrier plays an essential role in absorbing nutrients and preventing bacteria and toxins from the intestinal lumen [4, 5], and the intestine has been considered as the motor of multiple organ dysfunction syndromes during sepsis for a long time, which allows the translocation of bacteria and toxins from the intestinal lumen to circulation after damage [6], and in turn aggravates the progress of sepsis, while the effective treatment is absent [7].

Microvesicles (MVs) are natural membranous vesicles carrying a lot of biologically active molecules such as antigens, growth factors, mRNAs, and lipids, which are proved to participate in the regulation of various pathophysiological processes [8–10]. Stem cell-derived MVs are emerging therapeutic strategies for many diseases due to the MV-carried bioactive substances and the low immunogenicity [9–12]. For example, MSC-derived MVs (MMVs) were proved as an effective treatment in severe bacterial pneumonia by delivering keratinocyte growth factor to inflammatory cells [13]. MMVs were able to promote the recovery of acute kidney injury by improving the proliferation and inhibiting the apoptosis in tubular cells [14]. Studies also showed that stem cell-derived MVs could be used in the treatment of gastrointestinal diseases. MSC-derived extracellular vesicles could polarize macrophages and attenuated the colonic inflammatory response in inflammatory bowel disease and protected the function of the gut barrier by transferring metallothionein-2 [15]. Therefore, we hypothesized that MMVs might improve sepsis-induced intestinal barrier dysfunction, which might be related to the therapeutic proteins carried in MMVs.

Previous studies showed that MMVs usually exerted therapeutic effects via modulating genes and proteins in target cells [13, 14, 16–19], and recent studies suggested that MMVs might also improve the function of organelles as mitochondria. For example, MMVs could attenuate mitochondrial damage to alleviate inflammation in acute kidney injury [20], and improve mitochondrial fragmentation and ATP generation to promote the recovery of kidney function [21]. Mitochondria are important organelles in cells, which provide energy for various physiological processes [22, 23]. In addition to energy metabolism, mitochondria are also involved in a variety of pathophysiological processes, such as calcium ion homeostasis, cell apoptosis, and proliferation [22–24]. Under physiological circumstances, mitochondria are in a dynamic balance of continuous fission and fusion [22, 25], while the balance would be broken during sepsis, which manifests as excessive mitochondrial fission and insufficient mitochondrial fusion, along with the break of cristae and the decrease of mitochondrial quality [22, 25, 26]. The imbalance of mitochondrial dynamics could lead to the generation of ROS and the release of mtDNA, which is a major reason for cellular dysfunction [25–27]. Therefore, we further speculate that MMV might restore the function of intestinal epithelial cells to improve the intestinal barrier function by improving mitochondrial dynamic balance after sepsis. To test this hypothesis, MMVs were used to treat sepsis rats and intestinal epithelial cells (IEC-6), and the role and the underlying mechanism of MMVs on mitochondrial dynamic balance and intestinal barrier function were observed in the present study.

## Materials and methods

### Ethics approval and sepsis procedure

The animal use and operation procedures were approved by the Ethics Committee of the Army Medical Center (Daping Hospital, Army Medical University, Chongqing, China, No. DHEC-2012-069). Sprague-Dawley (SD) rats of either sex (weighting 190–215g) were provided by the Animal Center of the Army Medical Center. The cecal ligation and puncture (CLP) procedure was induced as described previously [28, 29]. Briefly, rats were anesthetized by sodium pentobarbital (25mg/kg, IP), and a 2-cm incision was made along the linea alba, then the end of the cecum was exposed and ligated, and the ligated cecum was punctured by a triangular pyramid, and the above operations were carried out under aseptic conditions. After the operation, rats were allowed water and food *ad libitum*.

### Cell culture and agents

MSCs were extracted from the tibia and femoral bone marrow of weaned SD rats (weighting 45–75g, 3–4 weeks old). In detail, the tibia and femur were collected after rats were sacrificed under aseptic conditions. Then, the bones were washed 5 times with sterile PBS, and the bone marrow was extracted by flushing the bones with 6mL MesenCult^TM^ Expansion Kit media (Stemcell Technologies, Canada), supplied with 80U/ml penicillin (Gibco, America) and 2mM L-Glutamine (Stemcell Technologies, Canada) [30]. Then, the mixed medium was cultured at 37°C and 5% CO_2_, and cells at 3–5 passages were used to harvest MMVs. IEC-6 was purchased from ATCC (America) and was cultured in DMEM high glucose medium with 10% fetal bovine serum (Gibco, America) and 80U/ml penicillin (Gibco, America). Chondrocytes were obtained from the cartilage of the hip and knee joints of SD rats (5–6 weeks old). In detail, the cartilages were washed by sterile PBS and digested with 0.25% trypsin (Gibco, America) for 40min, then digested by 0.2% collagenase II (Sigma, America) for 2h at 37°C. Then, the mixed medium was centrifuged by 200*g* for 10min, and the sediment was resuspended by 5mL DMEM-F12 medium supplemented with 5% Fetal bovine serum (Gibco, America), 100U/ml penicillin (Gibco, America) [31].

### MMV harvest

MMVs were harvested from the supernatant of cultured cells as described previously [28]. Briefly, when MSCs grew to 75% confluence, cells were washed with PBS and cultured in exosome-free medium with 1% serum for 48h. Subsequently, the supernatant was collected and centrifuged at 500*g* for 20min to remove dead cells (4°C), then the supernatant was centrifuged at 2000*g* for 20min to remove cell debris (4°C), then the supernatant was centrifuged at 20,000*g* for 70min, and the pellet was washed once with PBS and resuspended, finally stored at −80°C for further use.

### Imaging flow cytometry

MMVs were characterized by flow cytometry as described previously [28]. Briefly, CD29 (BD, America), CD73 (BD, America), CD90 (BD, America), CD105 (BD, America), CD105 (BD, America), and AnnexinV (BD, America) were applied to identify MMVs, where CD45 was the negative marker for MMVs and the others were positive markers for MMVs. Different antibodies were added to MMVs according to the experiment design, and MMVs were stained for 30min at RT in dark. After being washed once with PBS, MMVs were measured by the Amnis ImageStream MK II (IS^X^) (Millipore, America), and the detection magnification is × 40 and the flow rate is medium.

### Transmission Electron Microscopy (TEM)

MMVs were characterized by TEM as described previously [28]. Briefly, the MMV pellet was fixed by 2.5% glutaraldehyde for 24h at 4°C. After being washed by 0.1M PBS, MMVs were post-fixed by 1% OsO4 for 1h. Then, MMVs were dehydrated with gradient ethanol, embedded with TAAB 812, and cut into 100-nm sections. Finally, MMVs were observed by JEM 1400 TEM (JEOL, Japan).

### Dynamic Light Scattering (DLS)

DLS analysis was performed as described previously [28]. Briefly, MMVs were analyzed by Zetasizer Nano ZS (Malvern, America) by 633nm He-Ne laser at RT.

### Measurement of EB leakage

After the rats were sacrificed, about 5.5-cm length of jejunum was collected and the intestinal lumen was gently rinsed 4–6 times by PBS, then 1ml of 0.6% EB solution (Sigma, America) was added into the intestinal lumen. Then, both ends of the intestine were ligated and incubated in PBS for 30min at 37°C. After being washed 4–6 times, the intestine was dried for 20h (55°C) in the oven. The dried intestine was weighed and cut into pieces, and subsequently dissolved by 1ml formamide (Sigma, America). The EB absorbance was measured by Microplate Reader (Thermo, America). The EB leakage was calculated by the od-value of EB (A.U.)/tissue weight (g).

### Wet weight/dry weight ratio

After the rats were sacrificed, about 5.5-cm length of jejunum was collected and the intestinal lumen was gently rinsed 4–6 times by PBS. Then, the intestine was weighed for the wet weight. Afterward, the intestine was dried for 20h (55°C) in the oven and was weighed for the dry weight.

### Elisa (D-lac, TNFα, Zonulin, aspartate)

The rats were anesthetized and 4ml of blood was collected from the abdominal aorta, then the blood sample was centrifuged by 2000*g* for 10min, and the supernatant was used to detect D-lac (Jiancheng, China), TNFα (Jiancheng, China), and Zonulin (Biocompare, America) by the corresponding Elisa kit. The IEC-6 was collected and used to detect the concentration of aspartate by Aspartate Assay kit (Abcam, America), and the protein concentration of IEC-6 was measured by the BCA kit (Thermo, America). The concentration of aspartate was calculated by aspartate content (nmol)/protein content (mg).

### HE staining

After the rats were sacrificed, 1.5-cm length jejunum was collected and gently washed, then the intestine was fixed with 4% paraformaldehyde for 24 h, dehydrated with gradient ethanol, embedded with paraffin, and cut into pieces. Then, the sections were stained by HE staining and observed by Leica microscope (Leica, Germany).

### Western blot

The protein of the intestine and IEC-6 were extracted by RIPA buffer, and the protein extracts were separated by SDS-PAGE and transferred to the PVDF membrane. Then, the corresponding antibodies were incubated according to the experiment design, and the Odyssey Clx (Li-Cor, America) was used to measure the protein level. PCNA and Tomm20 were purchased from CST (America), and mfn1, mfn2, GOT1, PGC-1α, and β-actin were purchased from Abcam (America) [28, 32].

### Transepithelial Electric Resistance (TEER) and BSA leakage

The TEER was measured as described previously and briefly described as follows [28]. IEC-6 was seeded (1×10^5^ per well) on the upper layer of Transwell 6-well plate (0.4μm, Coring, America), and different treatment was added according to the experiment design when the cells grew to full confluence (LPS: 5μg/ml, MMV: 2×10^6^/ml). Then the Voltohmmetre (World Precision Inc, America) was used to detect the TEER every 0.5h. The BSA leakage of IEC-6 was measured after TEER measurement. The upper insert was added with FITC-BSA (10μg/ml), then 200μl medium was collected from the lower insert every 10min with the same volume of fresh medium supplementing into the upper insert. The BSA leakage of IEC-6 was calculated as (B10+B20+B30+B40+B50+B60)/total fluorescence intensity, and B*x* represented the fluorescence intensity at *x* min.

### CCK8 analysis

IEC-6 was seeded (1×10^3^ per well) in the 96-well plate (Corning, America), and the medium was changed to 100μl serum-free medium when cells grew to 75% confluence, then 10μl CCK8 detection solution (Dojindo Laboratories, Japan) was added per well and the plate was incubated at 37°C for 1.5h. The absorbance was measured at 450nm by Microplate Reader (Thermo, America). The CCK8 value was calculated as (*A*_sample_ − *A*_blank_)/(*A*_control_ − *A*_blank_).

### Migration of IEC-6

IEC-6 was seeded (1×10^5^ per well) in the 6-well plate (Corning, America), and the medium was changed to serum-free medium when cells grew to full confluence. A sterile pipette tip was used to scratch a line in the center of per well, and the cell morphology was observed at 0h and 24h by Leica microscope (Leica, Germany).

### Immunofluorescence (IF)

The immunofluorescence was measured as described previously with some minor modifications [28]. Briefly, after IEC-6 was stimulated with corresponding treatment, the cells were fixed by 4% paraformaldehyde for 20min, ruptured by 0.3% Triton-100 for 9min, and blocked by goat serum (Beyotime, China), and ZO-1 (CST, America) primary antibody, secondary antibody (Thermo, America), and DAPI (Thermo, America) were incubated. ROS, Tunel, mitochondria, and JC-1 (ΔΨm) were stained according to the instruction of each kit. ROS kit was purchased from Jiancheng (China), Tunel kit was purchased from Roche (Germany), Mitochondrial kit was purchased from Roche (Germany), and JC -1 kit was purchased from Beyotime (China). Confocal imaging was performed by Leica SP5-II confocal microscope (Germany).

### MMV absorption of intestine and IEC-6

The measurement of MMV absorption was performed as described previously [28]. Briefly, MMVs were stained by PKH-26 (Sigma, America) at 37°C for 0.5h, and MMVs were washed and resuspended. Then, PKH-26-labeled MMVs were injected into rats (I.V.), the intestinal tissue was collected for frozen section by freezing microtome (Leica, Germany) 8h later, and the frozen section were stained by DAPI (Thermo, America) and analyzed by Leica SP5-II confocal microscope (Germany). For cell experiments, IEC-6 were co-incubated with PKH-26-labeled MMVs for 8h, then were stained by DAPI, and analyzed by confocal microscope.

### ATP measurement

IEC-6 cell extracts were collected after appropriate stimulation, then were detected by ATP detection kit (Beyotime, China). Meanwhile, the protein concentration was measured by the BCA kit (Thermo, America). The ATP content was presented by ATP concentration (nmol) / protein content (μg).

### IEC-6 Oxygen Consumption Rate (OCR)

OCR of IEC-6 was performed as described previously [33]. Briefly, cell extracts were collected and centrifuged at 200*g* for 10min, then the sediment was resuspended with buffer (137mM NaCl, 0.7mM Na2HPO4, 5mM KCl, 25mM Tris-HCl, 1mM EDTA, pH7.45, at28°C), and the OCR was measured by Clark electrodes (Strathkelvin, Scotland). The unit of OCR was nM/min/10^6^ cells.

### Harvest of modified MMVs

To generate modified MMVs, MSCs were transfected with the corresponding adenovirus. Briefly, the medium of MSCs was changed to serum-free Opti-MEM when cells grew to 75% confluence; mfn2 overexpressing adenovirus, mfn2 shRNA adenovirus, mfn2 mock adenovirus, PGC-1α overexpressing adenovirus, PGC-1α siRNA adenovirus, and PGC-1α mock adenovirus were used to treat MSCs (MOI 50) for 24h according to the experiment design (Obio Technology, China); then the medium was changed to exosome-free medium with 1% serum; and the modified MMVs were harvested at 48h later. The target sequences were as follows:

PGC-1α siRNA, 5′-CCTGGACACAGACAGCTTT-3′. Mfn2 shRNA, Forward: 5′-CcggGGACCCAGTTACTACAGAATTCAAGAGATTCTGTAGTAACTGGGTCCTTTTTTg-3′, Reverse: 5-aattcaaaaaaGGACCCAGTTACTACAGAATCTCTTGAATTCTGTAGTAACTGGGTCC-3′.

### Statistical analysis

Data from animal studies were repeated at least eight independent experiments, and data from cell studies were repeated at least five independent experiments, and data from one representative experiment were shown and presented as mean±SD. Statistical analysis was performed by SPSS v19.0. Differences among groups were analyzed by one-way ANOVA test. The survival rate was analyzed by Kaplan-Meier survival analysis. Values of *p*<0.05 were considered significant.

## Results

### 1. MMVs improved sepsis-induced intestinal barrier dysfunction

#### The intestinal barrier function was severely impaired after sepsis

**Fig. 1:**
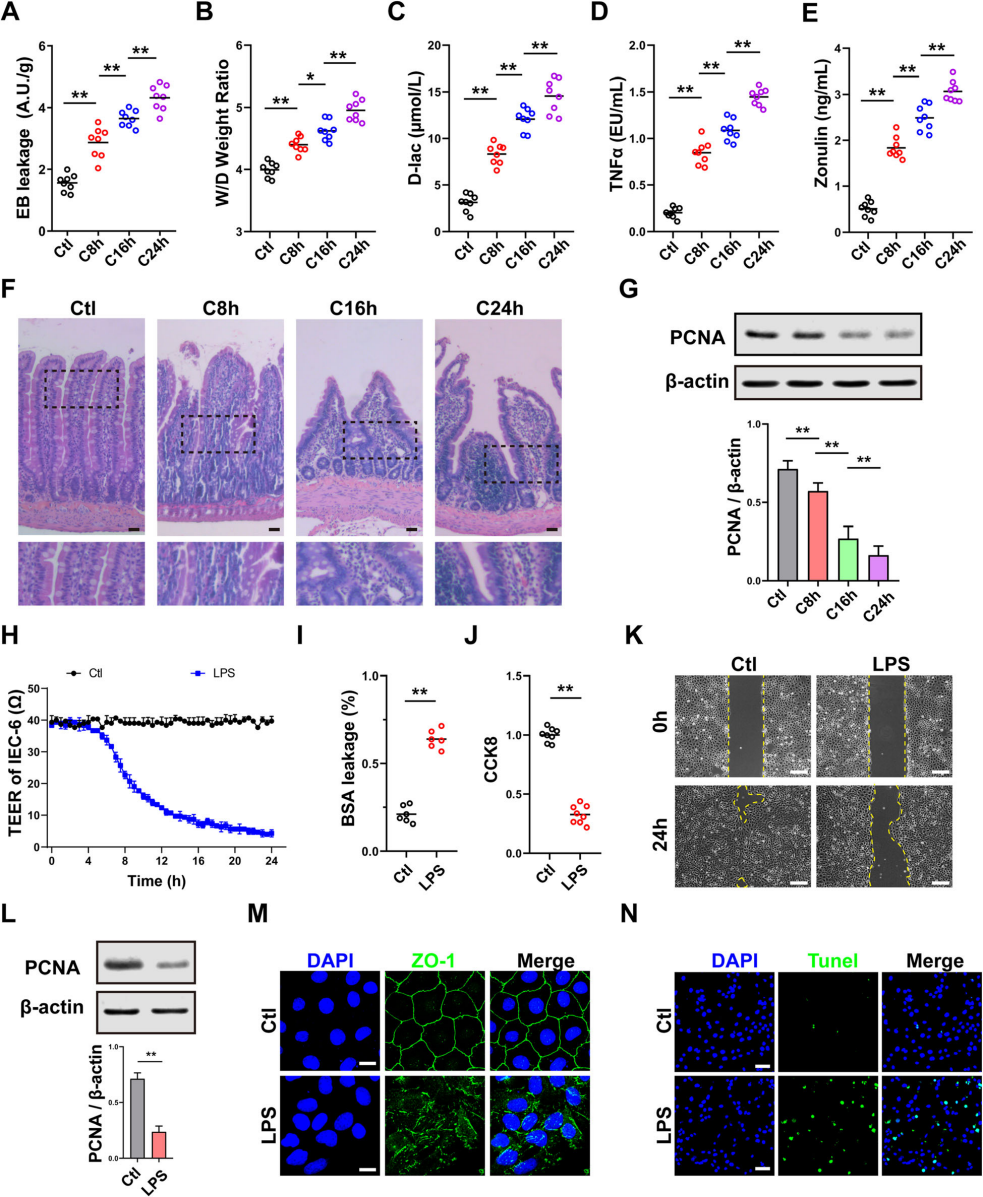
The intestinal barrier function was impaired after sepsis. **a** EB leakage of intestine at different times after sepsis (*n*=8). **b** Wet weight to dry weight ratio of intestine at different times after sepsis (*n*=8). **c** Blood D-lac level at different times after sepsis (*n*=8). **d** Blood TNFα level at different times after sepsis (*n*=8). **e** Blood Zonulin level at different times after sepsis (*n*=8). **f** Representative microphotographs of HE staining in intestine. The scale bar represents 100μm. **g** The relative expression of PCNA in intestine at different times after sepsis. β-actin was used as the internal reference (*n*=5). **h** TEER of IEC-6 after LPS stimulation (*n*=5). **i** BSA leakage of IEC-6 after LPS stimulation (*n*=6). **j** CCK8 proliferation assay of IEC-6 (*n*=6). **k** Representative microphotographs of the scratch migration assay and the scratched area were shown. The scale bar represents 100μm. **l** The relative expression of PCNA in IEC-6 after LPS stimulation. β-actin was used as the internal reference (*n*=5). **m** Representative IF microphotographs of ZO-1 in IEC-6 after LPS stimulation. The scale bar represents 20μm. **n** Representative IF microphotographs of Tunel assay in IEC-6. The scale bar represents 50μm. Ctl, normal control group; C8h, 8h after CLP group; C16h, 16 h after CLP group; C24h, 24h after CLP group. **P < 0.01, *P < 0.05

Data showed that the EB leakage of the intestine was significantly increased after sepsis, and the increment rate was 55%, 72%, and 127% at 8h, 16h, and 24h, respectively, which was aggravated in a time-dependent manner (Fig. 1a). Under physiological conditions, the wet/dry weight ratio of intestine was 4.1, and it was increased in a time-dependent manner after sepsis after sepsis (Fig. 1b). Besides, the blood D-lac and TNFα concentration also increased in a time-dependent manner after sepsis (Fig. 1c, d). HE results showed that the intestine villus was intact, the columnar epithelium was arranged neatly, and the brush border was smooth in normal rats, while the structure of the intestine was impaired after sepsis, which was characterized by columnar epithelium necrosis, intestine villus ruptured, inflammatory cell, and red blood cell infiltration (Fig. 1f). Zonulin is a typical biomarker for intestinal hyperpermeability [34], which can represent the degree of intestinal barrier dysfunction. The results showed that the blood Zonulin was significantly increased in a time-dependent manner after sepsis (Fig. 1e). The expression of PCNA in the intestine was significantly decreased after sepsis (Fig. 1g), indicating that the proliferation ability was significantly decreased.

LPS-stimulated IEC-6 was used to verify the physiopathologic changes in the intestine, and the results showed that the TEER of IEC-6 was significantly decreased after LPS stimulation (Fig. 1h), and the BSA leakage in IEC-6 was increased at the same time (Fig. 1i). The proliferation ability of IEC-6 was decreased significantly after LPS stimulation (Fig. 1j), along with the decrease of the expression of PCNA (Fig. 1l). Scratch assay showed that the migration ability of IEC-6 was decreased after LPS stimulation (Fig. 1k). Meanwhile, the ZO-1 barrier was impaired after LPS stimulation (Fig. 1m), and the apoptosis of IEC-6 was significantly increased (Fig. 1n). These results suggested that the intestinal barrier function was significantly impaired after sepsis.

#### MMV improved the intestinal barrier function after sepsis

**Fig. 2:**
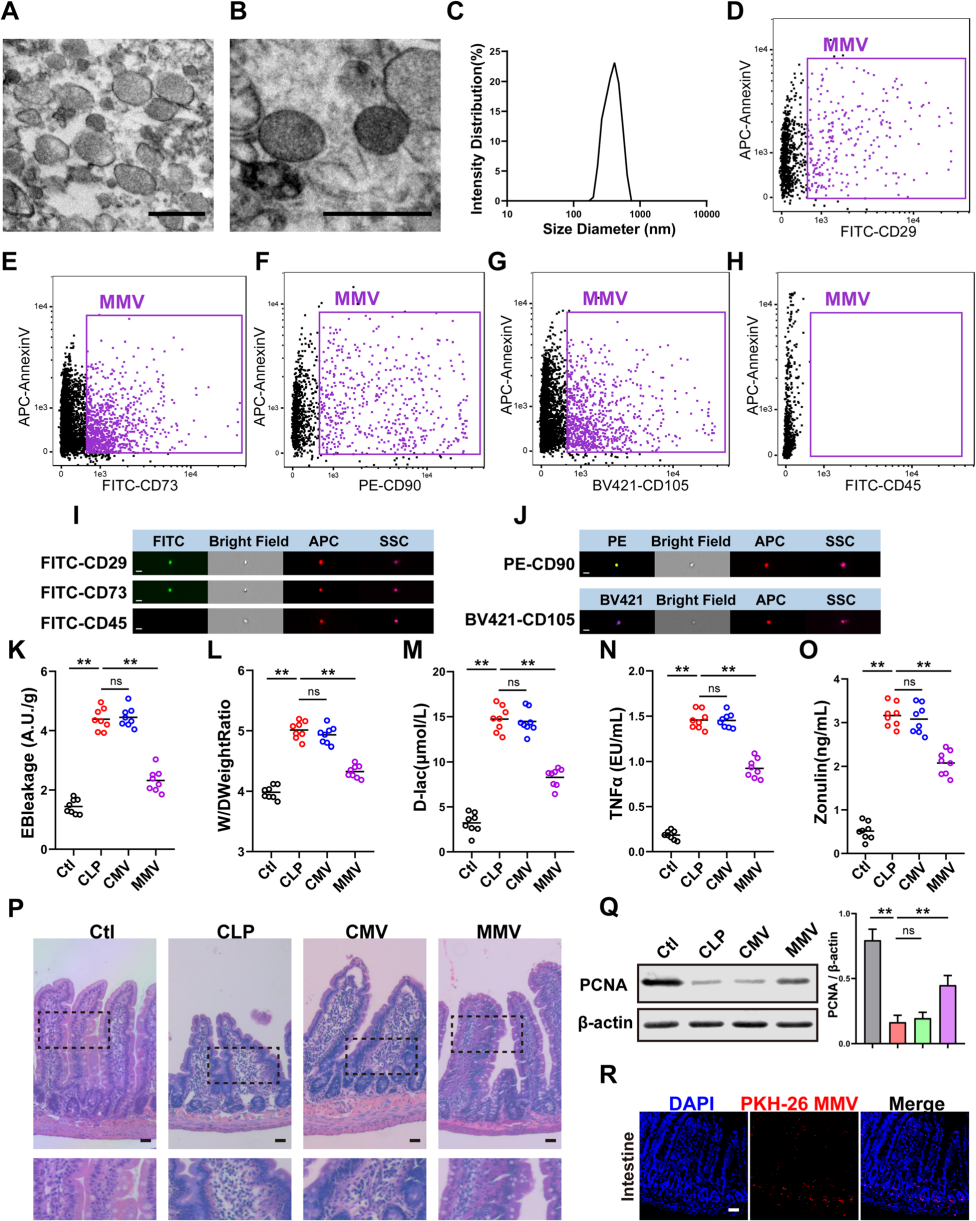
MMVs improved the intestinal barrier function after sepsis. **a**, **b** Representative TEM microphotographs of MMVs. The scale bar represents 500nm. **c** DLS analysis of the diameter distribution of MMVs (*n*=3). **d**–**h** Imaging flow cytometry analysis of MMV markers. **d**–**g** were the positive markers for MMVs, and **h** was the negative marker for MMVs. **i**, **j** Representative imaging flow cytometry microphotographs of MMVs. The scale bar represents 500nm. **k** Effect of MMVs on EB leakage of intestine (*n*=8). **l** Effect of MMVs on wet weight to dry weight ratio (*n*=8). **m** Effect of MMVs on blood D-lac level (*n*=8). **n** Effect of MMVs on blood TNFα level (*n*=8). **o** Effect of MMVs on blood Zonulin level (*n*=8). **p** Representative microphotographs of HE staining in intestine treated by MMVs. The scale bar represents 100μm. **q** The relative expression of PCNA in intestine treated by MMVs. β-actin was used as the internal reference (*n*=5). **r** Representative microphotographs of the endocytosis of PKH-26-labeled MMVs in intestine. The scale bar represents 100μm. Ctl, normal control group; CLP, sepsis group; CMV, CMV-treated group; MMV, MMV-treated group. **P < 0.01

Firstly, MMVs were harvested and identified. TEM results showed that MMVs exhibited typically spherical or quasi-spherical morphology and biomolecular membrane structure (Fig. 2a, b), with a diameter distribution of 100–1000nm. DLS results showed that the diameter of MMVs ranges from 168 to 942 nm (Fig. 2c), which was consistent with the results of electron microscopy. Imaging flow cytometry showed that MMV expressed MV-positive marker AnnexinV and MSC-positive marker CD29, CD73, CD90, and CD105, and did not express MSC-negative marker CD45 (Fig. 2d–h). Meanwhile, the microphotographs of imaging flow cytometry also exhibited the spherical structure of MMVs (Fig. 2i, j); these results indicated that the purity of MMVs was very high.

Then, 2×10^7^ MMVs were infused to sepsis rats, and chondrocyte-derived MVs (CMVs) were used as control. Based on this, the same dose of CMVs was used as a control group, and the same volume PBS (300μl) was infused to the normal and sepsis rats at the same time. The results showed that CMVs had no effect on EB leakage of intestine, while MMVs significantly reduced the EB leakage (Fig. 2k). Meanwhile, MMVs significantly improved the wet/dry weight ratio of the intestine in contrast to CLP group (Fig. 2l) and decreased the blood D-lac, TNFα, and Zonulin concentration (Fig. 2m–o), while CMVs had no effect at the same time (Fig. 2k–o). HE results showed that MMVs improved the morphology of intestinal villus, repaired columnar epithelium, and reduced the inflammatory cell and red blood cell infiltration (Fig. 2p). MMVs also increased the expression of PCNA in the intestine while CMVs had no effect (Fig. 2q). To investigate the MMV-absorption of the intestine, PKH-26-labeled MMVs were infused intravenously to sepsis rats, and the results showed that a large amount of scattered red fluorescence was observed in the intestine (Fig. 2r), indicating that MMVs could be effectively absorbed by intestine.

**Fig. 3:**
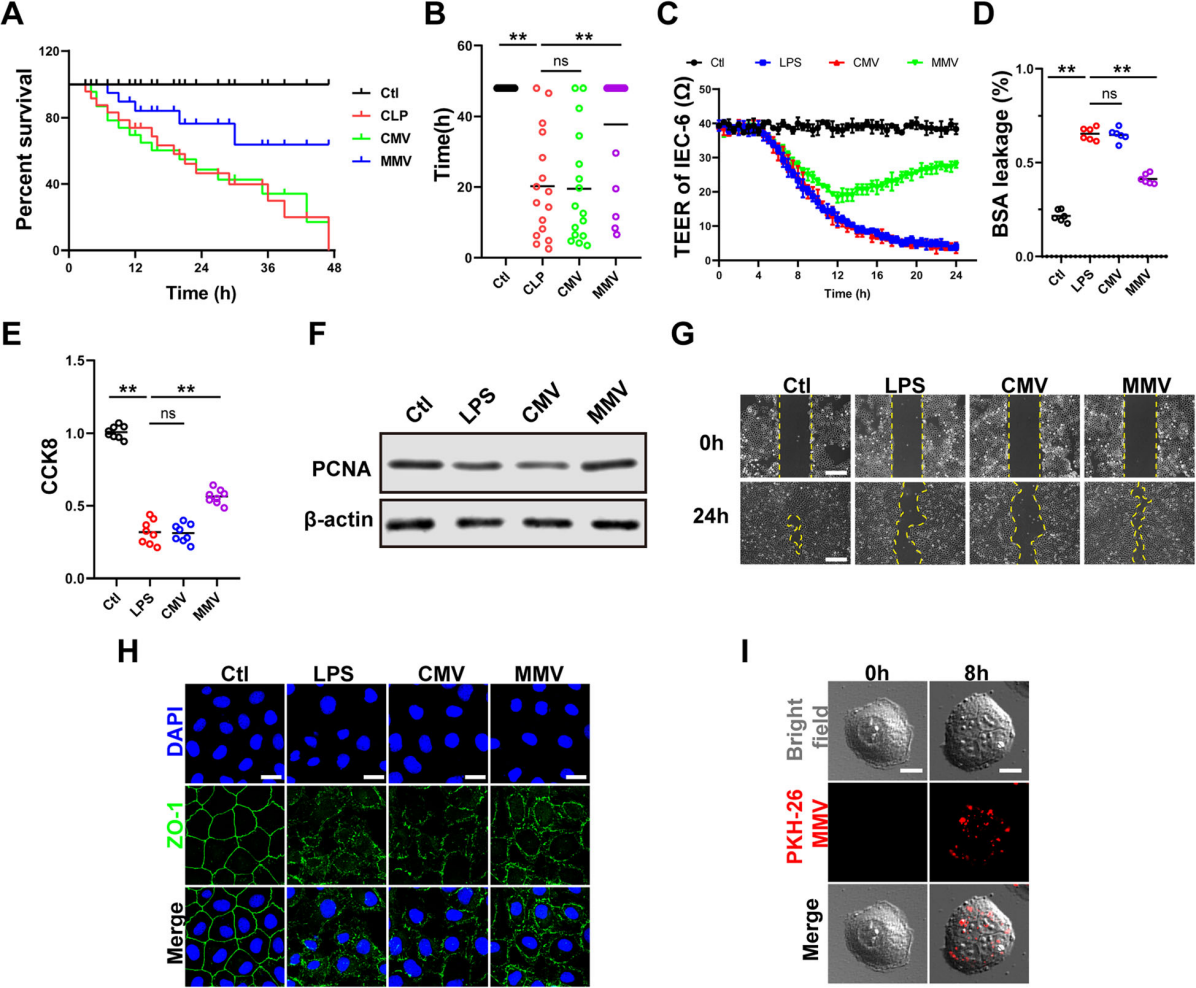
MMVs improved the survival rate of sepsis rats and the function of IEC-6. **a**, **b** Animal survival curve and survival time (*n*=16). **c** Effect of MMVs on TEER of IEC-6 (*n*=5). **d** Effect of MMVs on BSA leakage of IEC-6 (*n*=6). **e** Effect of MMVs on CCK8 proliferation assay of IEC-6 (*n*=6). **f** The relative expression of PCNA in IEC-6 treated by MMVs. β-actin was used as the internal reference (*n*=5). **g** Representative microphotographs of scratch migration assay and the scratched area of IEC-6 treated by MMVs. The scale bar represents 100μm. **h** Representative IF microphotographs of ZO-1 in IEC-6 treated by MMVs. The scale bar represents 20μm. **i** Representative microphotographs of the endocytosis of PKH-26-labeled MMVs in IEC-6. The scale bar represents 10μm. Ctl, normal control group; LPS, LPS-stimulated group; CMV, CMV-treated group; MMV, MMV-treated group. **P < 0.01

Further results showed that MMVs significantly improved the survival rate of sepsis rats and prolonged the survival time, with the mortality reducing from 56 to 25% (Fig. 3a), and the survival time improving from 22.8 to 38.5h (Fig. 3b).

#### MMV improved the proliferation and barrier function of IEC-6 after LPS stimulation

To investigate the effect of MMVs on IEC-6, 2×10^6^ MMVs were incubated with LPS-stimulated IEC-6. MMVs significantly increased the TEER (Fig. 3c) and decreased the BSA leakage (Fig. 3d) compared with the LPS group, while CMVs had no effect at the same time (Fig. 3c, d). CCK8 results showed that MMVs improved the proliferation ability of IEC-6 (Fig. 3e). The expression of PCNA was also significantly increased by MMVs treatment (Fig. 3g). In addition, the migration rate of IEC-6 was improved by MMVs (Fig. 3f). Meanwhile, MMVs also improved the expression and the integrity of ZO-1 (Fig. 3h). The co-incubation of PKH-26-labeled MMVs and IEC-6 showed that MMVs could be efficiently absorbed by IEC-6 (Fig. 3i). These results suggested that MMVs could significantly improve the proliferation and barrier function of IEC-6.

### 2. MMVs restored the proliferation and barrier function of IEC-6 by improving mitochondrial dynamic balance

#### MMVs improved mitochondrial dynamic balance

**Fig. 4:**
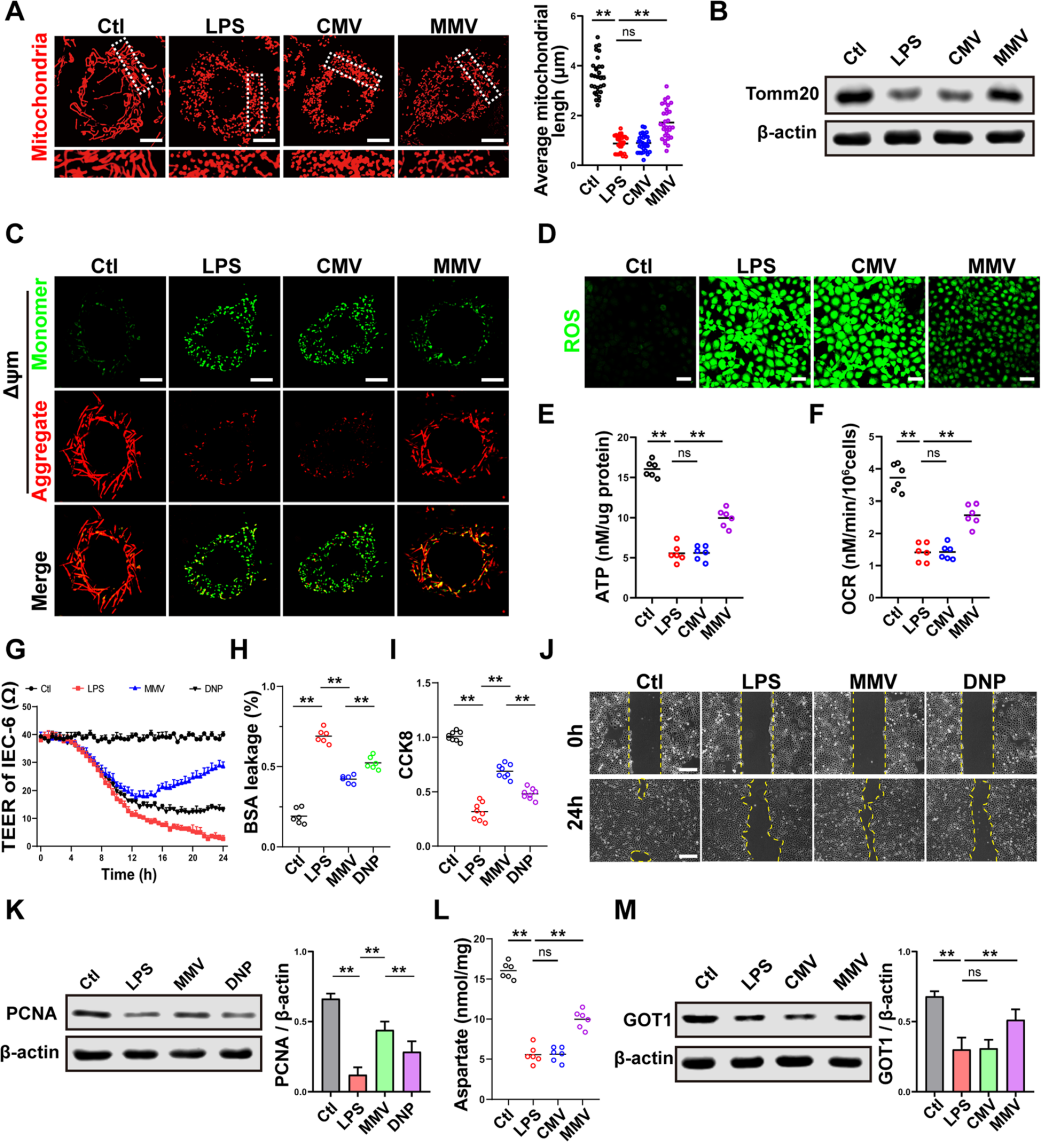
MMVs improved mitochondrial function of IEC-6. **a** Representative microphotographs of mitochondria labeled with mitotracker-Red and the average mitochondria length in 30 cells. The scale bar represents 10μm. **b** The relative expression of Tomm20 the in IEC-6 treated by MMVs. β-actin was used as the internal reference (*n*=5). **c** Representative microphotographs of ΔΨm in IEC-6 treated by MMVs. The scale bar represents 10μm. **d** Representative microphotographs of ROS in IEC-6 treated by MMVs. The scale bar represents 50μm. **e** ATP concentration in IEC-6 treated by MMVs (*n*=6). **f** OCR in IEC-6 treated by MMVs (*n*=6). **g** The influence of uncoupling agent DNP on the effect of MMVs on TEER of IEC-6 (*n*=5). **h** The influence of DNP on the effect of MMVs on BSA leakage (*n*=6). **i** The influence of DNP on the effect of MMVs on CCK8 proliferation assay (*n*=6). **j** The influence of DNP on the effect of MMVs on scratch migration assay. The scale bar represents 100μm. **k** The influence of DNP on the effect of MMVs on the relative expression of PCNA. β-actin was used as the internal reference (*n*=5). **l** The concentration of aspartate in IEC-6 treated by MMVs (*n*=6). **m** The relative expression of GOT1 in IEC-6 treated by MMVs. β-actin was used as the internal reference (*n*=5). Ctl, normal control group; LPS, LPS-stimulated group; CMV, CMV-treated group; MMV, MMV-treated group; DNP: MMV+DNP group. **P < 0.01

Mitochondria are in a dynamic balance of continuous fission and fusion, and the disorder of mitochondrial dynamic balance is an important cause of cell dysfunction [35–37]. Therefore, the effect of MMVs on mitochondrial dynamic balance was observed. The results showed that mitochondria exhibited an extended tubular rod-like network and were evenly distributed in normal IEC-6, and the morphology appeared to be fragmented after LPS stimulation (Fig. 4a). To further clarify the morphological changes of mitochondria, 30 cells were randomly selected from each group, and the average length of mitochondria in each cell was calculated [35]. The results showed that the average length of mitochondria in the normal group was 3.52μm, and it was decreased to 0.65μm in LPS group. CMVs had no effect on the morphology and average length of mitochondria (Fig. 4a), while MMVs restored the morphology of mitochondria, and increased the average length of mitochondria to 2.31μm (Fig. 4a). WB results showed that the expression of Tomm20, representing the mitochondrial quality, was decreased after LPS stimulation, and it was restored by MMVs (Fig. 4b). These results suggested that MMVs could improve mitochondrial dynamic balance of IEC-6.

#### MMVs improved the oxidative phosphorylation (OXPHOS) and metabolism of mitochondria in IEC-6

The imbalance of mitochondrial dynamics can cause the increase of membrane permeability and mitochondrial dysfunction, with the loss of mitochondrial membrane potential (ΔΨm), resulting in the production of a large amount of ROS [22, 23, 38]. Confocal results showed that MMVs significantly restored the LPS-induced decrease of ΔΨm in IEC-6 (Fig. 4c), and significantly decreased the ROS production of IEC-6, which showed that MMVs could reduce the oxidative stress caused by LPS (Fig. 4d). Mitochondrial dysfunction could lead to the dysfunction of the electron transport chain and the inhibition of OXPHOS, leading to energy metabolism disorders [39, 40]. Thus, we speculate that the therapeutic effect of MMVs on mitochondrial function might be related to the OXPHOS. Our study showed that MMVs significantly increased ATP production and improved the energy metabolism in IEC-6 (Fig. 4e). The oxygen consumption rate (OCR) in IEC-6 was impaired after LPS stimulation, and MMVs significantly improved the OCR (Fig. 4f).

#### MMVs restored the proliferation and barrier function of IEC-6 by improving the OXPHOS and metabolism of mitochondria

We further used the uncoupling agent 2,4-dinitrophenol (DNP) to inhibit the mitochondrial OXPHOS in IEC-6. The results showed that MMVs decreased the BSA leakage and improved TEER and the proliferation and migration ability of IEC-6 (Fig. 4g–k), while DNP significantly inhibited the therapeutic effect of MMVs on TEER and BSA (Fig. 4g, h), and abolished the improving effect of MMVs on the proliferation and migration ability of IEC-6 (Fig. 4i–k). These results suggested that the improvement of MMVs was closely related to the OXPHOS of mitochondria. Once the OXPHOS is disordered, many metabolic processes of mitochondria will be affected such as the tricarboxylic acid cycle (TCA cycle), and cause the accumulation of various intermediate products such as citrate and succinate and the decrease of aspartate [23, 39]. Aspartate is crucial in the proliferation of cells; thus, we hypothesized that the therapeutic effect of MMVs on the proliferation and barrier function of IEC-6 was related to the production of aspartate [41, 42]. The results showed that the level of aspartate in IEC-6 was significantly decreased after LPS stimulation, and MMVs significantly increased the level of aspartate (Fig. 4l). The production of aspartate is regulated by GOT (glutamic-oxaloacetic transaminase) [43], and the application of MMVs significantly increased the expression of GOT1 (Fig. 4m). These results suggested that the therapeutic effect of MMVs on the proliferation and barrier function were closely related to the improvement of OXPHOS and metabolism of mitochondria.

### 3. The mechanism of MMVs improving the mitochondrial dynamic balance in IEC-6

#### MMVs delivered mfn2 to IEC-6 to promote the mitochondrial fusion

**Fig. 5:**
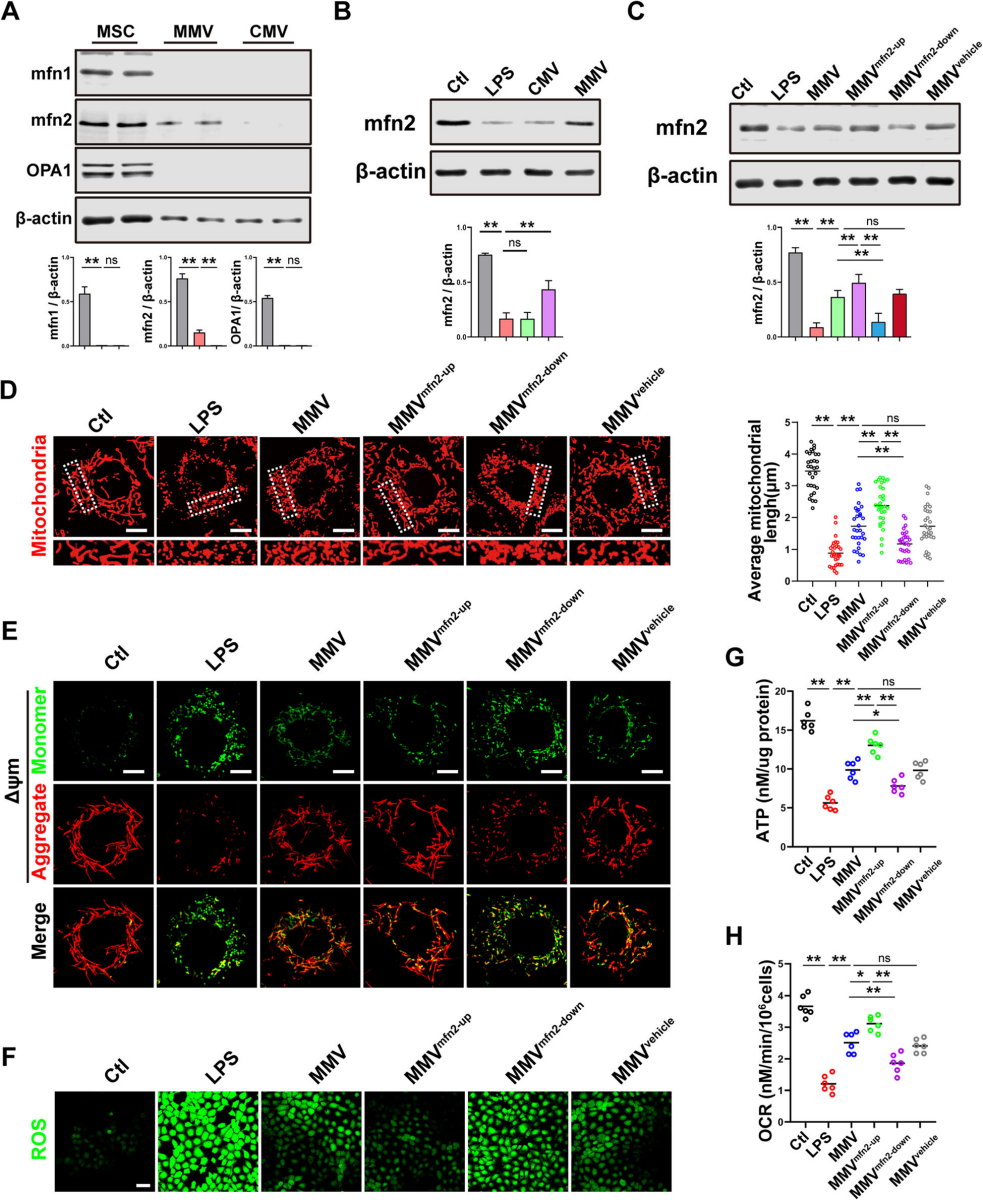
MMVs delivered mfn2 to IEC-6 to improve the mitochondrial function. **a** The relative expression of mfn1, mfn2, and OPA1 in MSCs, MMVs, and CMVs. β-actin was used as the internal reference (*n*=5). **b** The relative expression of mfn2 in IEC-6 treated by MMVs. β-actin was used as the internal reference (*n*=5). **c** The relative expression of mfn2 in IEC-6 treated by modified MMVs. β-actin was used as the internal reference (*n*=5). **d** Representative microphotographs of mitochondria treated by modified MMVs and the average mitochondria length in 30 cells. The scale bar represents 10μm. **e** Representative microphotographs of ΔΨm in IEC-6 treated by modified MMVs. The scale bar represents 10μm. **f** Representative microphotographs of ROS in IEC-6 treated by modified MMVs. The scale bar represents 50μm. **g** ATP concentration in IEC-6 treated by modified MMVs (*n*=6). **h** OCR in IEC-6 treated by modified MMVs (*n*=6). Ctl, normal control group; LPS, LPS-stimulated group; CMV, CMV-treated group; MMV, MMV-treated group; MMV^mfn2-up^, mfn2 upregulation in MMVs; MMV^mfn2-down^, mfn2 downregulation in MMVs; MMV^vehicle^, mfn2-mocktransfection in MMVs. **P < 0.01, *P < 0.05

Our above results showed that mitochondria in IEC-6 exhibited excessive fission and reduced fusion after LPS stimulation, while MMVs could improve the imbalance of mitochondrial dynamics, but the mechanism of MMVs in improving the mitochondrial dynamic balance is unclear. Therefore, we investigated whether MMVs could carry mitochondrial fusion proteins to promote the mitochondrial fusion in IEC-6. The results showed that mfn1, mfn2, and OPA1 were highly expressed in MSCs, while CMVs and MMVs did not carry mfn1 or OPA1 (Fig. 5a). However, mfn2 was expressed in MMVs but not in CMVs (Fig. 5a), which indicated that the promoting effect of MMVs on mitochondrial fusion might be related to MMV-carried mfn2. WB results showed that the expression of mfn2 in IEC-6 was decreased after LPS stimulation, while MMVs significantly increased the expression of mfn2 (Fig. 5b). In order to investigate whether the promoting effect of MMVs on mitochondrial fusion was caused by the MMV-carried mfn2, the mfn2-overexpressing adenovirus, mfn2 shRNA adenovirus, and mfn2 mock adenovirus were used to transfect MSCs, then the corresponding modified MMVs (MMV^mfn2-up^, MMV^mfn2-down^, MMV^vehicle^) were harvested and used to incubate with IEC-6 subsequently. The results showed that the expression of mfn2 in IEC-6 was further increased in the MMV^mfn2-up^ group compared with the MMV group, while the expression of mfn2 was decreased in the MMV^mfn2-down^ group, which was a little higher than the LPS group (Fig. 5c), suggesting that the overexpression of mfn2 in IEC-6 mainly resulted from the MMV-carried mfn2. Confocal observation showed that the mitochondrial morphology was further restored in the MMV^mfn2-up^ group compared with the MMV group, where the mitochondrial fragmentation was significantly reduced and cord-like mitochondria were further increased (Fig. 5d). However, the mitochondrial morphology was damaged in the MMV^mfn2-down^ group compared with the MMV group, indicating that MMVs delivered mfn2 to IEC-6 and subsequently improved mitochondrial dynamic balance by promoting mitochondrial fusion (Fig. 5d). Meanwhile, along with the improvement of mitochondrial dynamic balance, the mitochondrial function was also improved, which manifested the ΔΨm in IEC-6 was further improved in the MMV^mfn2-up^ group, while the ΔΨm in IEC-6 was decreased in the MMV^mfn2-down^ group (Fig. 5e). The production of ROS was further decreased in the MMV^mfn2-up^ group while the ROS was increased in the MMV^mfn2-down^ group (Fig. 5f). Further results showed that mfn2-upregulation in MMVs also increased the production of ATP and OCR compared with the MMV group, while mfn2-downregulation in MMVs inhibited the increasing effect of MMVs on ATP production and OCR (Fig. 5g, h). These results showed that MMVs could deliver mfn2 to IEC-6 and promote mitochondrial fusion, thereby improving mitochondrial function.

**Fig. 6:**
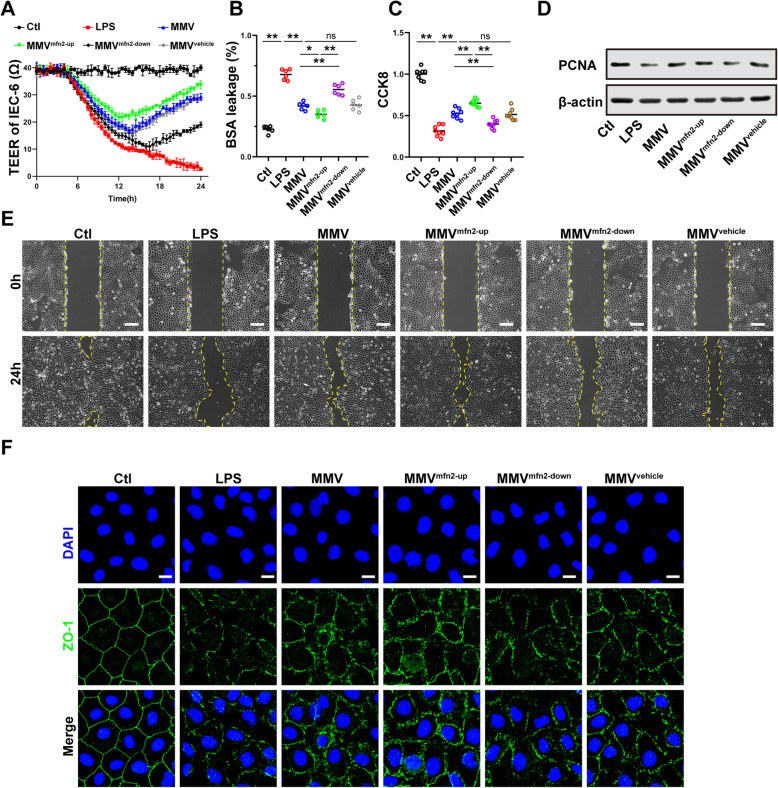
MMVs delivered mfn2 to IEC-6 to improve the proliferation and barrier function. **a** Effect of modified MMVs on TEER of IEC-6 (*n*=5). **b** Effect of modified MMVs on BSA leakage of IEC-6 (*n*=6). **c** Effect of modified MMVs on CCK8 proliferation assay of IEC-6 (*n*=6). **d** The relative expression of PCNA in IEC-6 treated by modified MMVs. β-actin was used as the internal reference (*n*=5). **e** Representative microphotographs of scratch migration assay and the scratched area of IEC-6 treated by modified MMVs. The scale bar represents 100μm. **f** Representative IF microphotographs of ZO-1 in IEC-6 treated by modified MMVs. The scale bar represents 20μm. Ctl, normal control group; LPS, LPS-stimulated group; CMV, CMV-treated group; MMV, MMV-treated group; MMV^mfn2-up^, mfn2 upregulation in MMVs; MMV^mfn2-down^, mfn2 downregulation in MMVs; MMV^vehicle^, mfn2-mocktransfection in MMVs. **P < 0.01, *P < 0.05

To further clarify the effect of mfn2 in MMVs on the barrier function of IEC-6, modified MMVs were incubated with IEC-6. Compared with the MMV group, mfn2-upregulation in MMVs enhanced the protective effect of MMVs on TEER and BSA leakage (Fig. 6a, b), while mfn2-downregulation in MMVs inhibited the effect of MMVs (Fig. 6a, b), and the vehicle-transfected MMVs showed no statistical difference (Fig. 6a, b). Besides, the proliferation and migration ability of IEC-6 was further improved in the MMV^mfn2-up^ group compared with the MMV group, while the proliferation and migration ability of IEC-6 was decreased in the MMV^mfn2-down^ group (Fig. 6c–e), and there was no statistical difference between the MMV^vehicle^ group and MMV group (Fig. 6c–e). Furthermore, the ZO-1 expression and integrity were also improved in the MMV^mfn2-up^ group compared with MMV group, while the integrity was impaired in the MMV^mfn2-down^ group (Fig. 6f).

#### MMVs delivered PGC-1α to IEC-6 to promote mitochondrial biogenesis

**Fig. 7:**
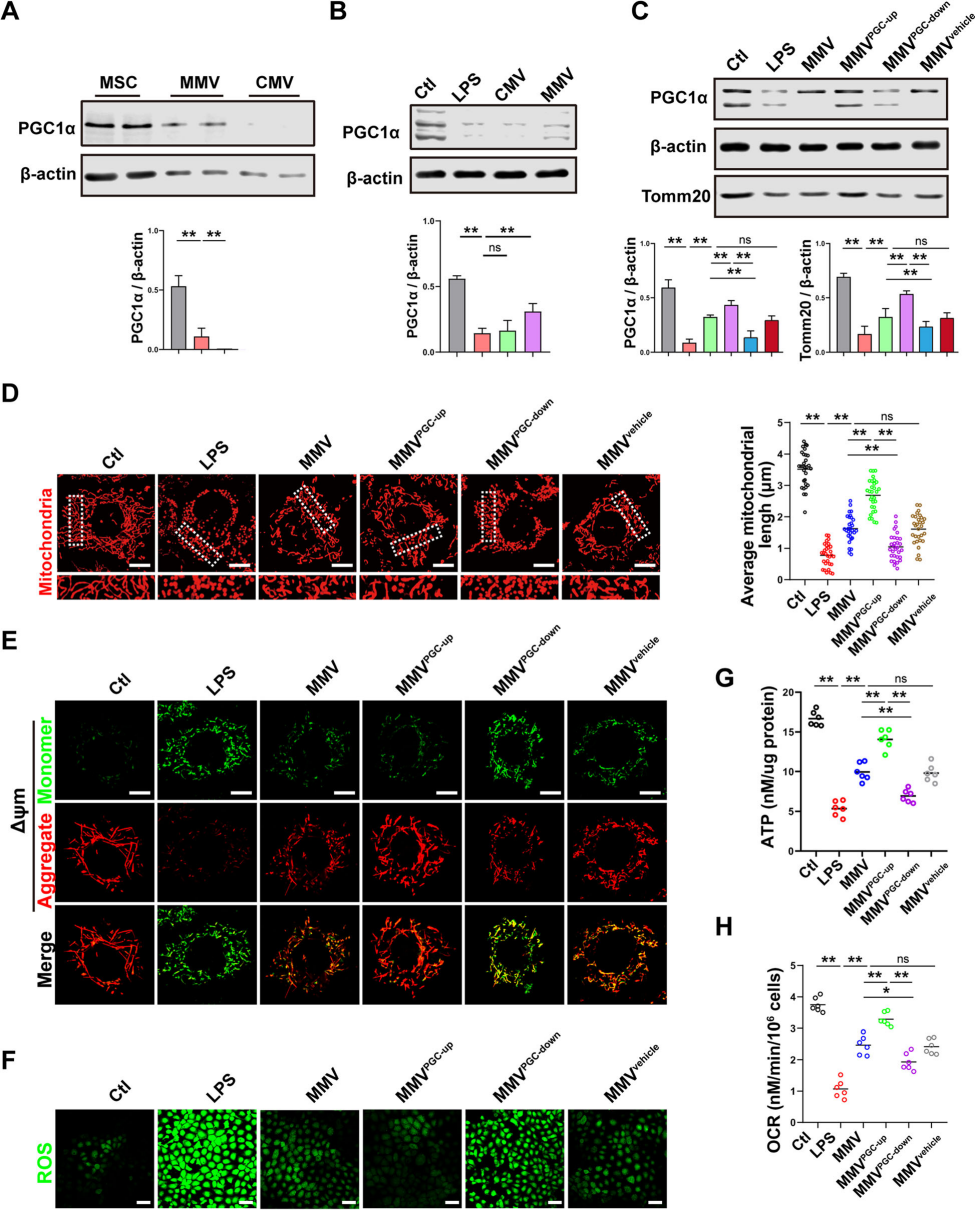
MMVs delivered PGC-1α to IEC-6 to improve the mitochondrial function. **a** The relative expression of PGC-1α in MSCs, MMVs, and CMVs. β-actin was used as the internal reference (*n*=5). **b** The relative expression of PGC-1α in IEC-6 treated by MMVs. β-actin was used as the internal reference (*n*=5). **c** The relative expression of PGC-1α in IEC-6 treated by modified MMVs. β-actin was used as the internal reference (*n*=5). **d** Representative microphotographs of mitochondria treated by modified MMVs and the average mitochondria length in 30 cells. The scale bar represents 10μm. **e** Representative microphotographs of ΔΨm in IEC-6 treated by modified MMVs. The scale bar represents 10μm. **f** Representative microphotographs of ROS in IEC-6 treated by modified MMVs. The scale bar represents 50μm. **g** ATP concentration in IEC-6 treated by modified MMVs (*n*=6). **h** OCR in IEC-6 treated by modified MMVs (*n*=6). Ctl, normal control group; LPS, LPS-stimulated group; CMV, CMV-treated group; MMV, MMV-treated group; MMV^PGC-1α-up^, PGC-1α upregulation in MMVs; MMV^PGC-1α-down^, PGC-1α downregulation in MMVs; MMV^vehicle^, PGC-1α mock-transfection in MMVs. **P < 0.01, *P < 0.05

Mitochondrial dynamic balance includes not only fission and fusion, but also biogenesis and mitophagy [22, 44]. The above results showed that MMVs could increase mitochondrial quantity; thus, we hypothesized that MMVs might deliver substances that are related to mitochondrial biogenesis. The results showed that PGC-1α was highly expressed in MSCs and MMVs, but not detected in CMVs (Fig. 7a). Co-incubation of MMVs and IEC-6 showed that MMVs significantly increased the expression of PGC-1α in IEC-6 after LPS stimulation (Fig. 7b). To investigate whether the high-expression of PGC-1α came from MMV-carried PGC-1α, modified MMVs (MMV^PGC-up^, MMV^PGC-down^, MMV^vehicle^) were harvested and incubated with IEC-6. The results showed that the expression of PGC-1α was higher in the MMV^PGC-up^ group than the MMV group, while the expression of PGC-1α was significantly decreased in the MMV^PGC-down^ group, suggesting that the increase of PGC-1α in IEC-6 was mainly from MMV-carried, rather than the endogenous generation of IEC-6 (Fig. 7c). In addition, the expression of Tomm20 was higher in the MMV^PGC-up^ group than the MMV group, while it was decreased in the MMV^PGC-down^ group (Fig. 7c), suggesting that the improvement of mitochondrial quantity resulted from MMV-carried PGC-1α. Confocal observation of the mitochondrial morphology showed that the mitochondrial morphology was further restored in the MMV^PGC-up^ group compared with the MMV group, while the mitochondrial network was damaged in the MMV^PGC-down^ group compared with the MMV group, indicating that MMVs could deliver PGC-1α to IEC-6 and improve mitochondrial dynamic balance by promoting mitochondrial biogenesis (Fig. 7d). Meanwhile, PGC-1α-upregulation in MMVs further enhanced the protective effect of MMVs on ΔΨm, ROS generation, ATP generation, and OCR in IEC-6 (Fig. 7e–h). The above results indicated that MMVs restored mitochondrial dynamic balance by promoting mitochondrial biogenesis, and subsequently improved mitochondrial function and energy metabolism.

**Fig. 8:**
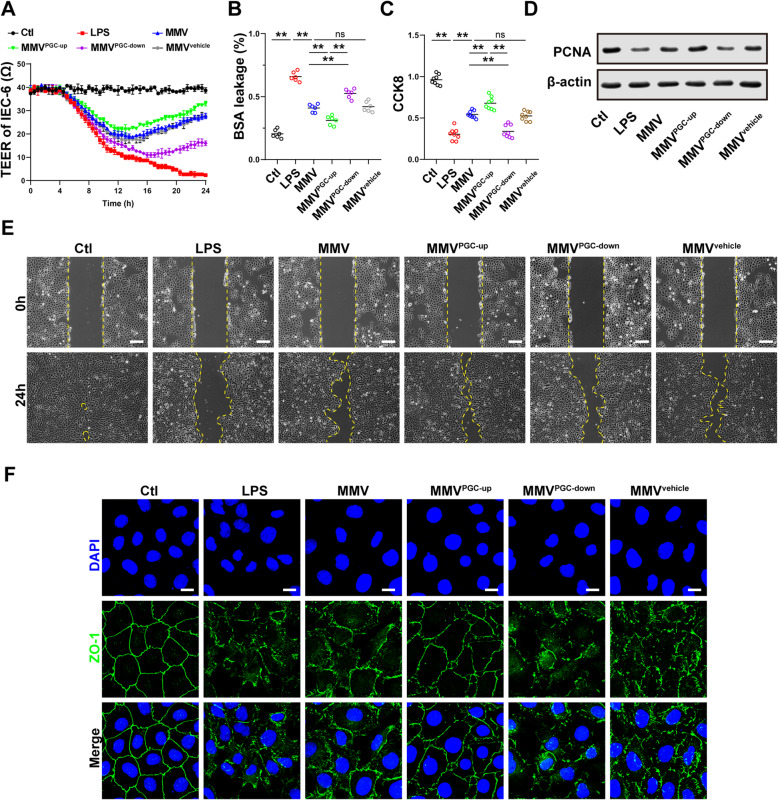
MMVs delivered PGC-1α to IEC-6 to improve the proliferation and barrier function. **a** Effect of modified MMVs on TEER of IEC-6 (*n*=5). **b** Effect of modified MMVs on BSA leakage of IEC-6 (*n*=6). **c** Effect of modified MMVs on CCK8 proliferation assay of IEC-6 (*n*=6). **d** The relative expression of PCNA in IEC-6 treated by modified MMVs. β-actin was used as the internal reference (*n*=5). **e** Representative microphotographs of scratch migration assay and the scratched area of IEC-6 treated by modified MMVs. The scale bar represents 100μm. **f** Representative IF microphotographs of ZO-1 in IEC-6 treated by modified MMVs. The scale bar represents 20μm. Ctl, normal control group; LPS, LPS-stimulated group; CMV, CMV-treated group; MMV, MMV-treated group; MMV^PGC-1α-up^, PGC-1α upregulation in MMVs; MMV^PGC-1α-down^, PGC-1α downregulation in MMVs; MMV^vehicle^, PGC-1α mock-transfection in MMVs. **P < 0.01

To further clarify the effect of PGC-1α in MMVs on the proliferation and barrier function of IEC-6, modified MMVs were incubated with IEC-6. The results showed that the PGC-1α upregulation enhanced the protective effect of MMVs on TEER and BSA leakage (Fig. 8a, b), while the PGC-1α-downregulation inhibited the effect of MMVs, and there was no statistical difference between the MMV^vehicle^ group and the MMV group (Fig. 8a, b). Moreover, the proliferation (Fig. 8c, d) and migration ability (Fig. 8e) and ZO-1 integrity (Fig. 8f) of IEC-6 were further improved in the MMV^PGC-up^ group, but decreased in the MMV^PGC-down^ group, and there was no statistical difference between the MMV^vehicle^ group and MMV group.

### MMVs delivered functional mitochondria to IEC-6 to improve the mitochondrial function of target cells

**Fig. 9:**
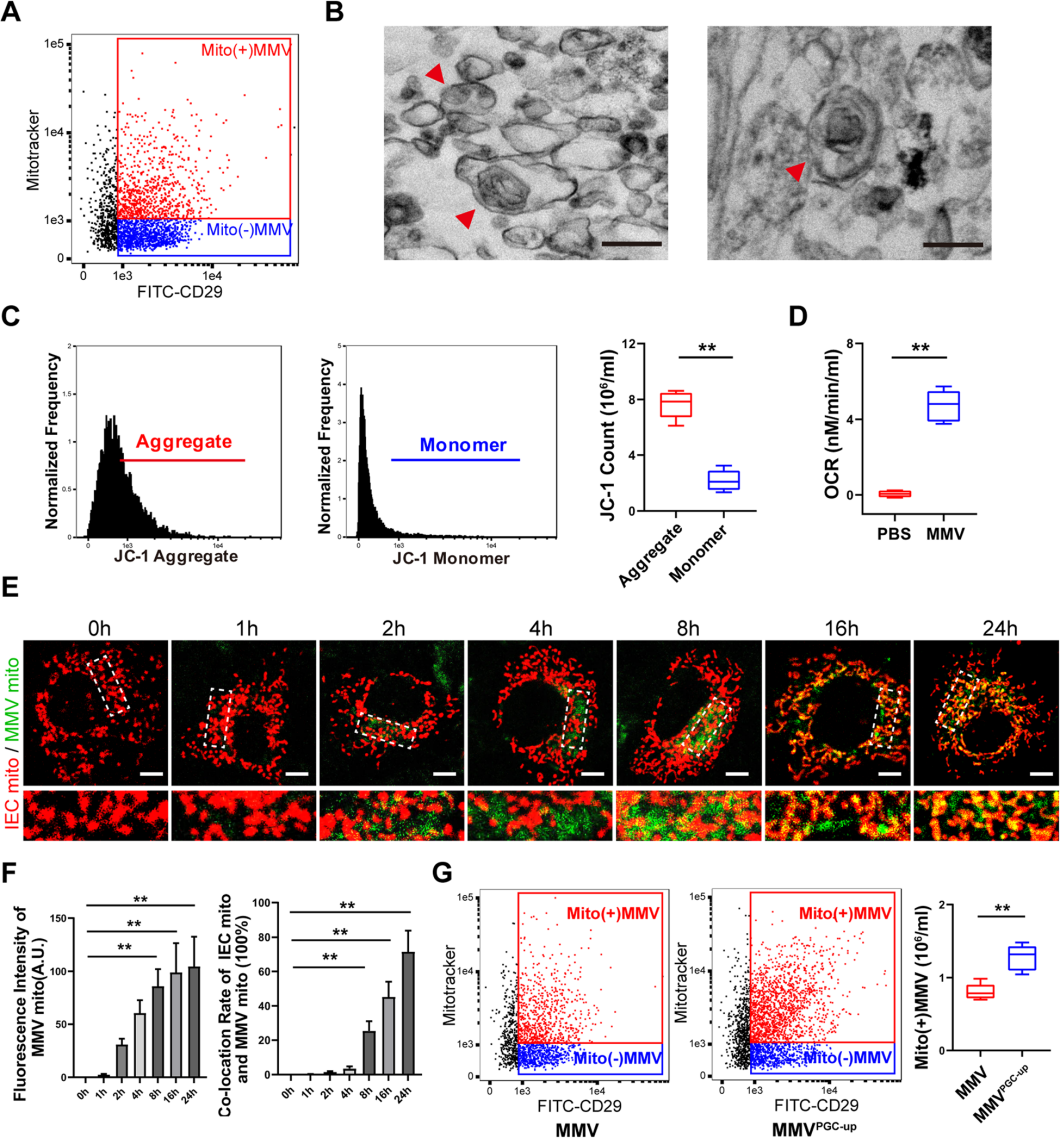
MMVs delivered functional mitochondria to IEC-6. **a** Flow cytometry analysis of mitochondria-positive MMVs detected by mitotracker-Red. **b** Representative TEM microphotographs of mitochondria-carried MMVs. The scale bar represents 500nm. **c** Flow cytometry analysis of ΔΨm in MMVs. **d** OCR analysis in MMVs (*n*=5). **e** Representative confocal microphotographs of the fusion of MMV-delivered mitochondria (Green pseudocolor) and the mitochondria in IEC-6 (Red pseudocolor). The scale bar represents 10μm. **f** The relative fluorescence intensity of MMVs and the co-location rate of two mitochondria in IEC-6 (*n*=5). **g** Flow cytometry analysis of mitochondria-positive MMVs in PGC-1α upregulated MMVs (*n*=5). **P < 0.01

Studies showed that in addition to biologically active substances, MVs might also carry small organelles [45–48]. Thus, we hypothesized that MMVs might carry functional mitochondria to improve the mitochondrial function in IEC-6 directly. To test this hypothesis, mitotracker-Red was used to stain MMVs, and imaging flow cytometry showed that the ratio of mitotracker-positive MMVs was 46% (Fig. 9a), indicating that nearly half of the MMVs carried mitochondria. TEM image showed that MMVs carried organelle-like structures containing cristae-like structures, which might be mitochondria (Fig. 9b). JC-1 staining showed that the red fluorescence (JC-1 Aggregates) was higher than the green fluorescence (JC-1 Monolayers) (Fig. 9c), which indicated that the mitochondria in MMVs had normal membrane potential. Detection of OCR in MMVs showed that the oxygen could be consumed normally (Fig. 9d). These results confirmed that MMVs carried functional mitochondria.

Then, we investigated whether functional mitochondria could be transferred to IEC-6. The RFP-mito plasmid was used to transfect MSCs and RFP-MMVs were harvested, then the RFP-MMVs (Green pseudocolor) were incubated mitotracker-Red-labeled IEC-6 (Red pseudocolor), and the confocal microphotograph was captured at different time (Fig. 9e). The results showed that only a small number of MMV-mitochondria were internalized into IEC-6 at 1h. The internalization of MMV-mitochondria was increased significantly at 2 h, but the co-location rate was only 3% (Fig. 9f). More MMV-mitochondria were internalized into IEC-6 at 4h, and the co-location rate was increased to 5%. Then, the MMV-mitochondria were internalized at a time-dependent manner, and the co-location rate also increased at a time-dependent manner, and the co-location rate reached 77% at 24h, which showed that the functional mitochondria in MMVs could be transferred into IEC-6, and fused with the mitochondria of IEC-6 and subsequently improved the function of mitochondria. Besides, we detected the ratio of mitochondria-positive MMVs after PGC-1α-upregulation, and the results showed that the PGC-1α-upregulation in MMVs also increased the mitochondria quantity in MMVs (Fig. 9g), which might be beneficial for the improvement of mitochondrial function in IEC-6.

## Discussion

Sepsis is one of the important causes of death for ICU patients [2]. The intestinal barrier function is disordered at the early stage of sepsis, while there is no effective treatment at present [6]. This study illustrated that MMVs could restore the intestinal barrier function and significantly improve the survival rate of sepsis rats. Further investigation found that MMVs could deliver mfn2, PGC-1α to IEC-6 to improve mitochondrial dynamic balance, and restore the OXPHOS and aspartate metabolism, thereby improving the proliferation and barrier function of intestinal epithelial cells and the intestinal barrier function. Meanwhile, MMVs may also deliver functional mitochondria to IEC-6 to restore the mitochondrial function of target cells directly. This study provides a new sight for the treatment in sepsis.

The intestinal epithelial cells are damaged and the intestinal permeability is increased at the early stage of sepsis, resulting in the translocation of intestinal flora and toxins from the intestine to the blood, which further exacerbates the organ dysfunction [4–6, 49]. At present, the clinical treatment strategies for intestinal hyperpermeability were mainly consist of anti-inflammation, antibiotics, fluid resuscitation, and supportive treatment, and the effect was limited. Recent studies also revealed that some emerging strategies were potentially therapeutic such as growth factors (epidermal growth factor, R-spondin) [4, 50], tight junction regulation (anti-MLCK) [51], neutralizing antibodies (anti-IL-1, anti-TNF-α, anti-IL-6) [52–54], and probiotics [4, 5], while the effect in clinic was not satisfactory. MVs were proved to be therapeutic in many diseases such as acute lung injury, acute kidney injury, and ischemia/reperfusion injury [13, 14, 55–57]; whether MVs were effective in intestinal barrier function after sepsis is obscure. The present study confirmed that MMVs could restore the sepsis-induced intestinal barrier dysfunction and promote the proliferation and migration ability intestinal epithelial cells. These findings provide a new sight for the treatment of intestinal disorder and mitochondrial dysfunction in critical illness such as sepsis.

Mitochondria are important organelles that provide energy for cell movement and play an important role in biological functions of cells [22, 44]. The present study found that sepsis could induce mitochondrial damage and the broken of mitochondrial dynamic balance. Under physiological circumstances, mitochondria are in a dynamic balance of fission and fusion [22, 23, 44]. During sepsis mitochondrial fission is excessive, while the fusion is deficient, which results in the disorder of mitochondrial functions [37]. Mitochondrial fusion is mediated by dynamin-related GTPase proteins, where mfn1, mfn2, and OPA1 play important roles [58]. Studies reported that mfn2 deletion could induce the mitochondrial fragmentation and promote the cell apoptosis by increasing the expression of Bax [26, 59]. This study found that the expression of mfn2 was significantly decreased after sepsis, resulting in insufficient mitochondrial fusion. MMVs can deliver mfn2 to IEC-6 and promote mitochondrial fusion and improve mitochondrial function. In addition, the fusion of mitochondrial is also related to mfn1 and OPA1, but the WB analysis revealed that MMVs did not carry mfn1 or OPA1, indicating that MSCs can selectively package proteins into MMVs, whether the mechanism remains to be further studied. Meanwhile, the present study showed that mitochondrial fission was decreased by MMVs; whether MMVs could interfere the expression of fission-related protein DRP1 needs to be further investigated.

Mitochondrial dynamic balance is a complicated process including mitochondrial fission, fusion, biogenesis, and mitophagy. The present study revealed that MMVs were also therapeutic in mitochondrial biogenesis by MMV-carried PGC-1α. PGC-1α plays a central role in mitochondrial biogenesis, which can activate a series of transcription factors such as PPAR, NRF1, ERRα, and other important proteins required for mitochondrial biogenesis [26, 60]. Our study found that MMVs could deliver mfn2 and PGC-1α to IEC-6 at the same time, and improved the mitochondrial fusion and biogenesis of target cells synergistically, and the downregulation of either mfn2 or PGC-1α in MMVs could inhibit the therapeutic effect of MMVs, which suggested that MMV-induced fusion and biogenesis by delivering functional proteins are crucial for mitochondrial dynamic balance and mitochondrial function. Interestingly, MMV^PGC-down^ could induce a slight increase in the expression of PGC-1α. Studies proved that PGC-1α could be regulated by multiple factors [61–65] as TNFα, AMPK, sirt1, Nitric oxide, miR-199, miR-211, miR-494, and MMVs were able to carry abundant proteins and miRNAs, which might be related to the increase of PGC-1α.

Mitochondrial dysfunction can induce damage to the electron transport chain and subsequently increase the production of ROS [23, 25]. MMVs can restore the mitochondrial dynamic balance, thereby significantly reducing the OXPHOS substrate of the electron transport chain and reducing ROS production and cell damage [26]. Mitochondrial OXPHOS may produce many intermediate metabolic products, which are not only involved in the electron transport chain and tricarboxylic acid cycle (TCA cycle) but also participate in many pathophysiological processes [39, 41]. The OXPHOS and TCA cycle are inhibited after sepsis, while the glycolysis is increased, resulting in the accumulation of massive intermediates such as citrate, succinate, and the decrease of NAD+, aspartate, etc. [27, 39]. Aspartate is the precursor of many purines and multiple amino acids, which are essential raw materials for cell proliferation [41, 42]. In the present study, we found that the concentration of aspartate in IEC-6 was decreased after LPS stimulation, and MMVs could restore the concentration of aspartate in IEC-6, which was related to the improvement of GOT1. Therefore, our results suggested that the therapeutic effect of MMVs on the proliferation and barrier function of IEC-6 was related to the improvement of OXPHOS and aspartate metabolism, but the detailed mechanism remains to be investigated. Besides, the accumulation of intermediary metabolites could participate in the inflammatory pathway to aggravate cell damage [66], whether MMVs could promote the clearance of these metabolites needs to be further determined.

Studies suggested that MSCs can deliver mitochondria to adjacent cells through intercellular TNT (Tunneling nanotube) or connexin 43, and improve energy metabolism in target cells [67, 68]. Besides, MMVs were reported to participate in the removal of damaged mitochondria in mother cells [69]. Whether MMVs carry functional mitochondria to improve mitochondrial dynamic balance is not known. Our study illustrated that MMVs could deliver healthy and functional mitochondria to IEC-6, and the transferred-mitochondria could be fused with the damaged mitochondria in IEC-6, then improved the mitochondrial function of the target cell directly. Although MMVs were able to consume oxygen, only part of MMVs carried intact mitochondria, and the effect of MMVs on mitochondrial dynamic balance mainly resulted from MMV-carried mfn2 and PGC-1α. Meanwhile, mfn2 carried by MMVs might be helpful for the fusion of the two kinds of mitochondria from MMVs and IEC-6 and synergistically improved the mitochondrial function in target cells, but it needs to be further confirmed. Moreover, our study found that the overexpression of PGC-1α in MSCs could increase the ratio of mitochondria-positive MMVs, and enable MMVs to carry more functional mitochondria to improve the proliferation and barrier function of target cells.

There are still some limitations and deficiencies in this study. Firstly, both MVs and exosomes were vesicles secreted from cells, and these two vesicles have similar characteristics and are beneficial for diseases [45, 46, 48]. Due to the large diameter, MMVs are more likely to carry mitochondria, so the present study was mainly focused on the role of MMVs, whether MSC-derived exosomes play a similar role in intestinal barrier dysfunction needs to be further studied. Secondly, the present study focused on the effect of MMVs on mitochondrial fusion and biogenesis; whether MMVs regulate mitochondrial fission and mitophagy remains to be determined. Thirdly, the coordinated effect of mfn2, PGC-1α, and functional mitochondria carried by MMVs on mitochondrial dynamic balance and intestinal and barrier function needs to be further investigated.

## Conclusions

**Fig. 10:**
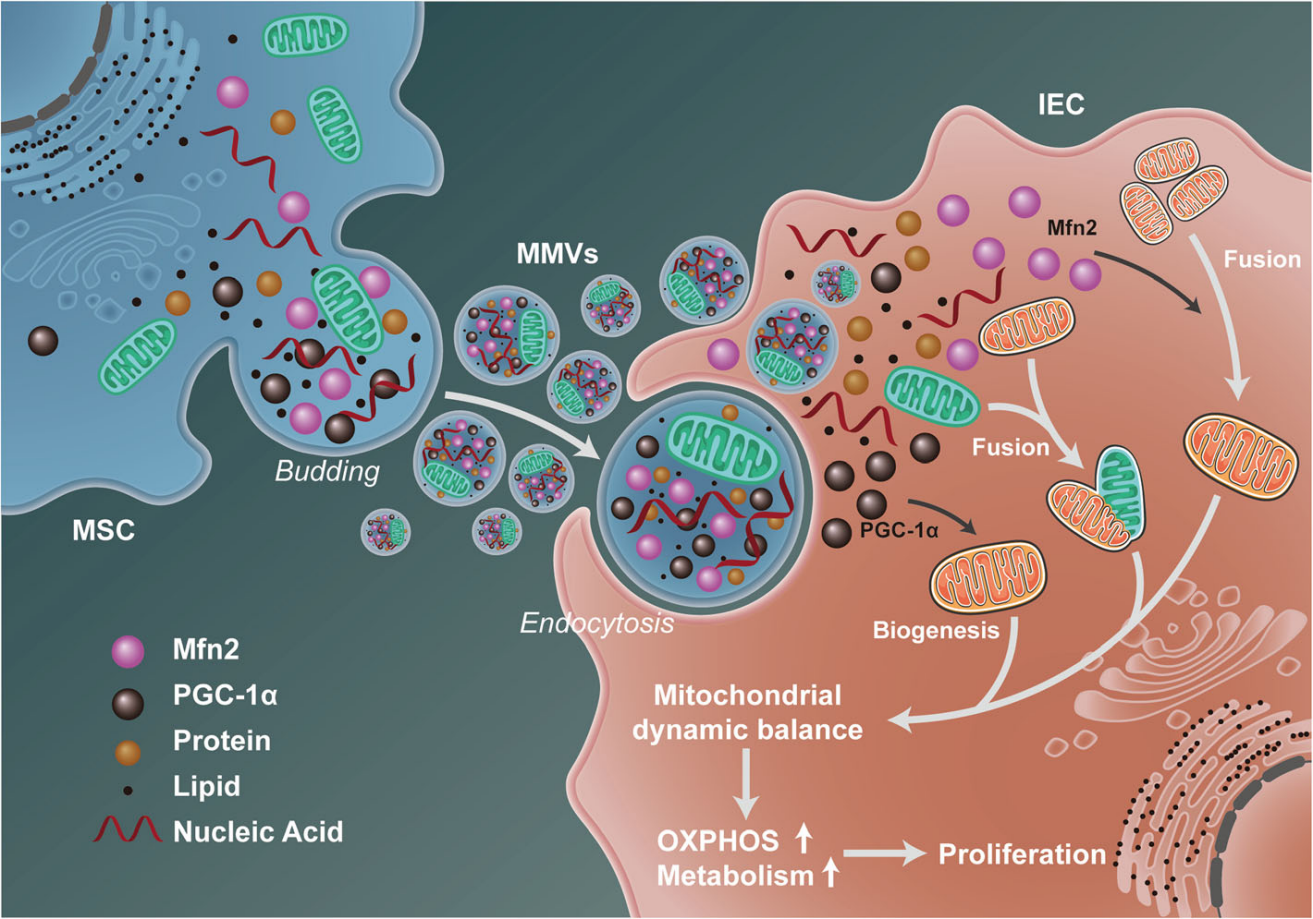
The schematic diagram for the mechanism of the therapeutic effect of MMVs on intestinal barrier function after sepsis. MMVs delivered mfn2, PGC-1α, and functional mitochondria to intestinal epithelial cells and synergistically restored the mitochondrial dynamic balance by promoting mitochondrial fusion and biogenesis, and subsequently improved mitochondrial oxidative phosphorylation and metabolism, thereby improving the intestinal barrier function

In summary, the present study innovatively illustrates that MMVs can deliver mfn2, PGC-1α, and functional mitochondria to intestinal epithelial cells, and synergistically improve mitochondrial dynamic balance of target cells after sepsis, and restore the mitochondrial function and intestinal barrier function, which was related to the improvement of mitochondrial OXPHOS and aspartate metabolism (Fig. 10). This study provides a new sight for the treatment of mitochondrial dysfunction and intestinal barrier dysfunction in critical illnesses such as sepsis.

## Data Availability

All of the data that support the findings of this study are available from the corresponding author Tao Li upon reasonable request.

## Abbreviations

CCK8: Cell counting kit-8
CLP: Cecal ligation and puncture
CMV: Chondrocyte-derived microvesicles
DLS: Dynamic light scattering
DNP: 2,4-Dinitrophenol
EV: Extracellular vesicles
GOT: Glutamic-oxaloacetic transaminase
IEC-6: Intestinal epithelial cell line
IF: Immunofluorescence
IRAK1: IL-1 receptor-related kinase 1
LPS: Lipopolysaccharide
MMV: Mesenchymal stem cell-derived microvesicles
MSC: Mesenchymal stem cells
MV: Microvesicles
OCR: Oxygen consumption rate
OXPHOS: Oxidative phosphorylation
SD: Sprague-Dawley
TCA: Tricarboxylic acid cycle
TEER: Transepithelial electric resistance
TEM: Transmission electron microscopy
TRAF6: TNF receptor-related factor 6
