# Correction: Mesenchymal stem cell-derived microvesicles improve intestinal barrier function by restoring mitochondrial dynamic balance in sepsis rats

**DOI:** 10.1186/s13287-024-03839-5

**Published:** 2024-07-11

**Authors:** Danyang Zheng, Henan Zhou, Hongchen Wang, Yu Zhu, Yue Wu, Qinghui Li, Tao Li, Liangming Liu

**Affiliations:** State Key Laboratory of Trauma, Burns and Combined Injury, Shock and Transfusion Department, Research Institute of Surgery, Daping Hospital, Army Medical University, Daping, Chongqing, 400042 People’s Republic of China

**Stem Cell Research & Therapy (2021) 12:299**

10.1186/s13287-021-02363-0

The article contains two errors in Figures 5 and 6:

1) In Figure 5, the immunofluorescence image of the MMV^vehicle^ group was inaccurate.

2) In Figure 6, the optical microscope image of the MMV^vehicle^ group was inaccurate.

The corrected figures can be viewed ahead in this correction article.

**Figure:**
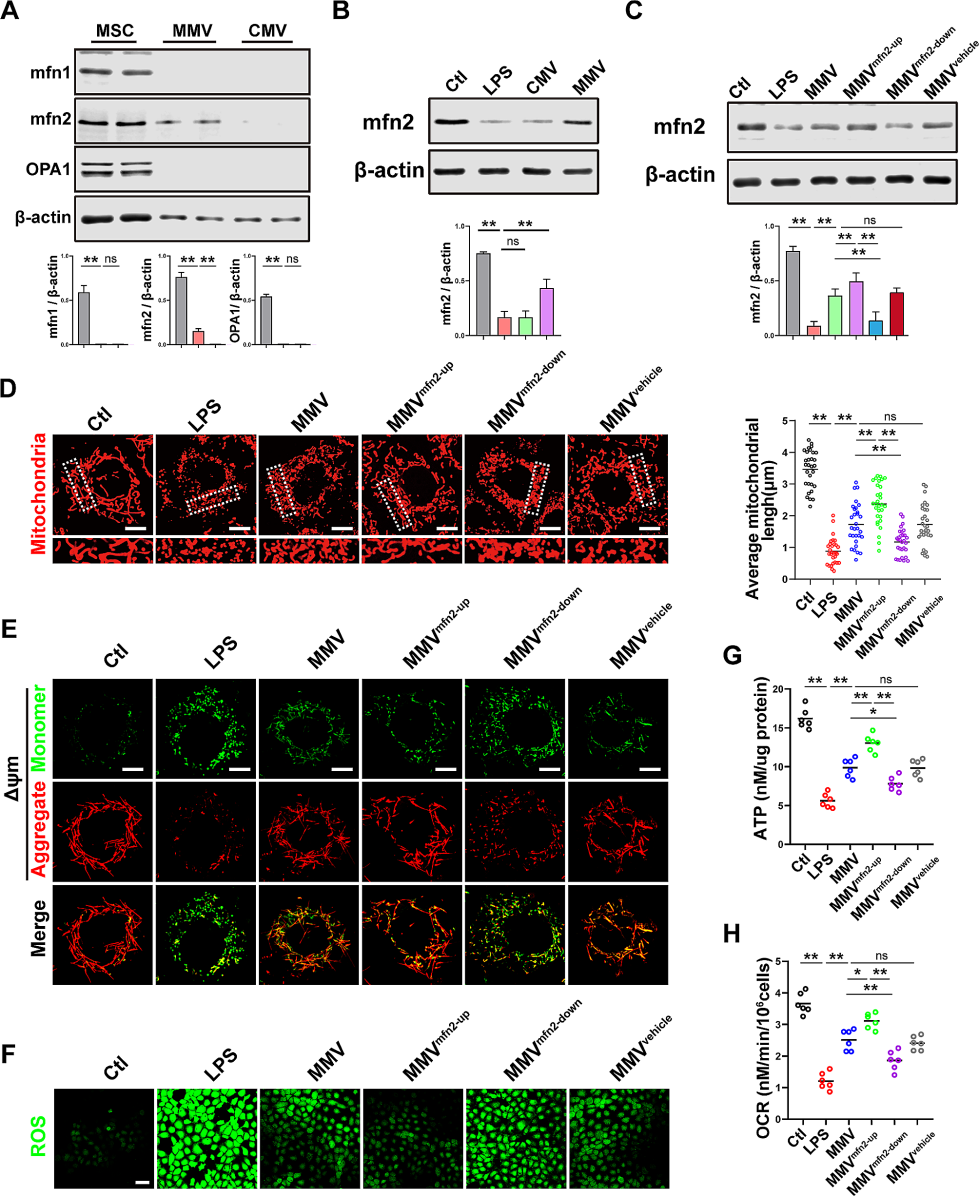
Fig. 5

**Figure:**
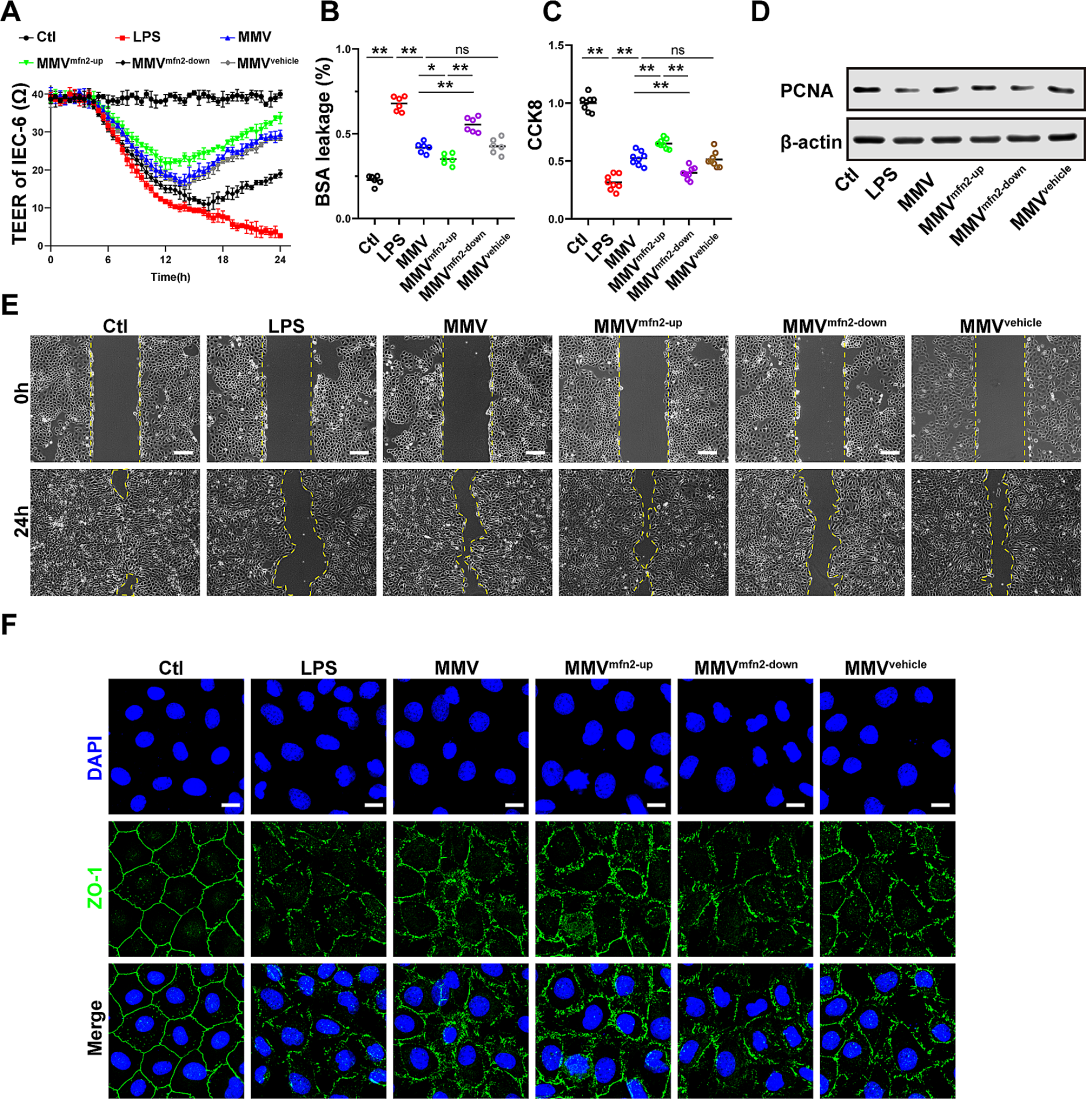
Fig. 6

